# Temporomandibular joint involvement in Juvenile Idiopathic Arthritis: reliability and validity of a screening protocol for the rheumatologist

**DOI:** 10.1186/s12969-015-0011-2

**Published:** 2015-05-07

**Authors:** Michel H Steenks, Gabriella Giancane, Rob RJ de Leeuw, Ewald M Bronkhorst, Robert JJ van Es, Ron Koole, H. Willemijn van Bruggen, Nico M Wulffraat

**Affiliations:** Department of Oral and Maxillofacial Surgery, Prosthodontics and Special Dental Care, University Medical Center Utrecht, Utrecht, The Netherlands; Pediatric Immunology, University Medical Center Utrecht, Utrecht, The Netherlands; Julius Center, University Medical Center Utrecht, Utrecht, The Netherlands; Department of Oral Function and Prosthetic Dentistry, College of Dental Science, Radboud university nijmegen medical center, Nijmegen, The Netherlands

**Keywords:** Juvenile idiopathic arthritis, Temporomandibular joint, Disease activity

## Abstract

**Background:**

In Juvenile Idiopathic Arthritis (JIA) the temporomandibular joint (TMJ) can be involved leading to pain, dysfunction and growth disturbances of the mandible and associated structures. There may be value to a three minute screening protocol allowing the rheumatologist to detect TMJ involvement systematically. Reliability and validity of the TMJ protocol for detecting TMJ co-morbidity were determined in 74 consecutive JIA patients.

**Methods:**

The assessments of the rheumatologist and of a reference examiner (RE) were compared and validity of the TMJ protocol was established using the disease activity (JADAS-27) as an external reference.

**Results:**

The internal consistency of the protocol was 0.73 (Cronbach’s alpha). The inter-examiner agreement between the rheumatologist and the RE varied between 0.25 and 0.87 (Cohen’s Kappa). Sensitivity and specificity, with the JADAS “3.8” indicating minimal disease activity, were 0.57 and 0.77 respectively. The area under the curve (AUC) was 0.70. A cut-off value of two positive items was found to be an optimal threshold to select the patients with likely TMJ involvement.

**Conclusions:**

The use of the protocol is feasible in everyday clinical practice. Reliability and validity aspects were satisfactory. The screening protocol for TMJ involvement provides the rheumatologist with systematic and focused TMJ information which relates to the JIA disease activity (JADAS-27).

## Background

Arthritis of the temporomandibular joint (TMJ) can be a manifestation of Juvenile Idiopathic Arthritis (JIA), with impact on the masticatory system [1,2]. It is described as occurring in all JIA subtypes, in recent onset as well as long-standing disease [3,4]. Failure to diagnose and treat TMJ arthritis may have severe consequences. When the TMJ is affected, children may have masticatory dysfunction, like pain on biting, chewing and yawning [5]. Mandibular growth can also be impaired and facial asymmetries may develop [6-9]. Therefore, the pediatric rheumatologist could find value in a brief screening TMJ examination protocol for patients with JIA, whereby TMJ dysfunction could be detected and managed. Based on the result of the assessments, the rheumatologist would be able to detect actual TMJ dysfunction and treat the underlying inflammation using aggressive management. Additionally, patients could be referred for a more comprehensive examination to a multidisciplinary team consisting of a maxillofacial surgeon, orthodontist, orofacial pain specialist and/or physical therapist for further diagnosis and management of possible complications of JIA. Pediatric rheumatologists may not be trained to perform a full diagnostic examination or do not have time in all consecutive patients. By completing the 3 minute screening protocol, all rheumatologists would be able to monitor TMJ status systematically rather than refrain from a full evaluation of the TMJ and related structures or examine on a non-systematic basis. Therefore a short examination protocol, derived from a validated model [10,11] was constructed, to be used by all rheumatologists in children and young adults with JIA.

The severity of TMJ involvement in JIA has been reported to be directly related to inflammatory variables: symptoms of TMJ dysfunction coincide with flares of disease activity [5]. Moreover, clinical and subjective orofacial involvement (e.g., temporomandibular signs and symptoms) appears to be related to disease activity in patients with JIA during the most recent two years, using the International League against Rheumatism classification (ILAR) [12]. For these reasons we used the JADAS-27 as an external clinical reference for validation of the TMJ protocol. The JADAS-27 reflects the overall JIA activity and does not include assessment of the masticatory system. A correlation between the total score of the TMJ protocol and the JADAS-27 may support its clinical utility.

Our aim was to test the TMJ examination protocol for internal consistency, reliability and concurrent validity against the JADAS-27 in a consecutive series of patients diagnosed with JIA.

## Methods

Research was in compliance with the Helsinki Declaration. The Medical-Ethics Committee of the UMC Utrecht approved all study procedures (12-604/C).

### Participants

Seventy eight consecutive patients with JIA, classified by the International League Against Rheumatism (ILAR) criteria [13], between 5 and 17 years of age, were selected for the study and recruited from the outpatient clinic of the pediatric department of rheumatology & immunology at the University Medical Center Utrecht, The Netherlands. They were requested to participate by mail two weeks before their consultation with the rheumatologist. Informed consent was obtained in 76 out of 78 patients. Reasons for non-participation were psychological due to fear of being examined by a dentist. Patients, either in remission or with active disease, were examined between December 2013 and January 2014. Those with dental problems, a history of facial trauma, or pre-existing cranio-maxillofacial disorder unrelated to JIA were excluded. Patient characteristics and subgroup diagnoses are described in Table 1.

**Table 1:** **Patient characteristics**

	**n**	**Mean**	**SD**	**Min - Max**
Examined	76			
Enrolled (F:M)	74 (2.4:1)			
Age (SD) yrs		11.9	3.8	
Age at onset (SD) yrs		7.5	3.9	
Disease duration (SD) yrs		4.5	3.4	
JIA diagnosis:				
Oligo ANA pos	17/23%			
Oligo ANA neg	15/20.3%			
Oligo extended	6 /8.1%			
Poly RF pos	16/21.6%			
Poly RF neg	8/10.8%			
Systemic JIA	8 (10.8)%			
ERA HLA B27 pos	3/4%			
Psoriatic Arthritis	1/1.4%			
Medication:				
NSAID	36/48.6%			
Systemic steroids	4/5.4%			
DMARDS	41/55.4%			
Biologics	22/29.7%			
Orthodontic tx	7/9.5%			
JADAS-27	74	3.6	5.8	0 – 37.5
MMO	74	45.3	7.7	25 – 60
MMO age ≤ 10	27	43.6	5.4	25 – 52
MMO age > 10	47	46.3	8.7	25 – 60
MMO ≤ 40	21	35.9	4.2	25 – 40
MMO ≤ 35	8	32.1	4.5	25 – 35
MMO ≤ 30	2	-	-	25 – 27

Number of patients referred to as n / % (unless indicated otherwise), and MMO as mm

Pt: patients; F: female; M: male; yrs: years, SD = standard deviation, n = number of patients. ANA: anti-nuclear antibodies; oligo: oligoarticular; poly: polyarticular; ERA: enthesitis-related arthritis; PsoA: psoriatic arthritis; NSAIDs: non-steroidal anti-inflammatory drugs; DMARDs: disease modifying anti-rheumatic drugs. Orthodontic tx: coincidental orthodontic treatment (patients being in treatment and wearing appliances not for JIA related TMJ issues). MMO: maximum mouth opening; JADAS-27: Juvenile Arthritis Disease Activity Score.

### Procedure

The pediatric rheumatologists were trained for the TMJ examination before the start of the project by a dentist (MHS) who is an orofacial pain and temporomandibular joint dysfunction specialist experienced with the clinical examination of the masticatory system and the examination of children with JIA. For this reason the examiner was considered as the Reference Examiner (RE). A two hour training session was sufficient to explain the aim of the study, the protocol and the clinical examination to the rheumatologists, who were then tested by the RE on four patients in the clinical setting. Written instructions regarding the protocol were handed out. Four weeks after the last training session the study started.

### Clinical examination

**Figure 1:**
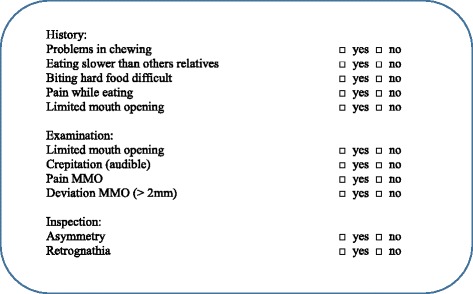
Format of the TMJ screening protocol.

**Figure 2:**
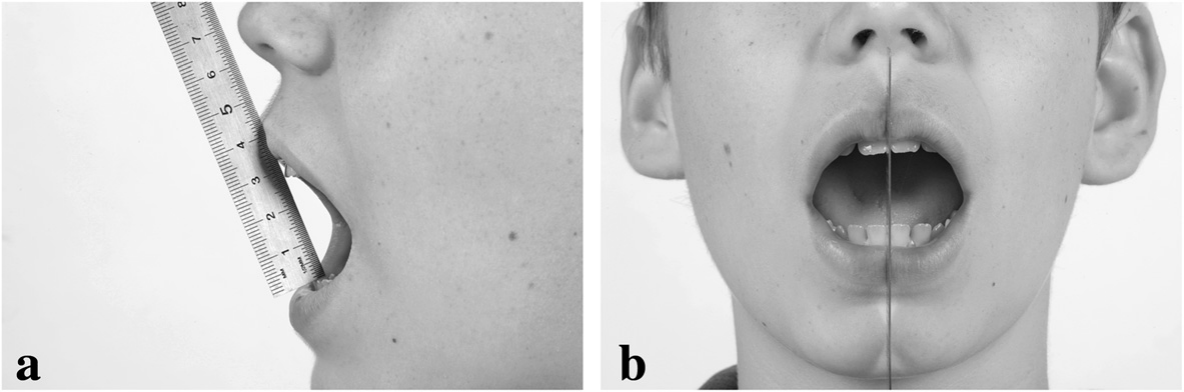
Measurement of parameters of maximum mouth opening (**a**) range of motion (**b**) lateral deviation on MMO > 2mm; in this illustration there is no lateral deviation.

On the day of consultation, the patient was first examined by the rheumatologist. Within 15–30 minutes, the patient was then examined by the dentist, blinded to the findings of the rheumatologist. The TMJ protocol consisted of five history related questions and six clinical examination items (Figure 1). History related items were: problems with chewing, eating more slowly than relatives, difficulties in biting and eating hard food, pain on chewing and limited mouth opening. The clinical examination included measuring maximum mouth opening (MMO) in millimeters between the incisal edges of the upper and lower front teeth with a metal ruler to the nearest mm (Figure 2a). Patients were encouraged to open actively as wide as possible. They were instructed not to pay attention to pain that might occur. The cut-off value for restricted mouth opening was ≤ 35 mm in ≤ 10 year olds and ≤ 40 mm in patients > 10 years [14]. The examiner assessed audible crepitation (without a stethoscope) during opening and closing, pain on maximum mouth opening, supplemented by inspection regarding asymmetry (chin, face) and mandibular retrognathia. Mandibular deviation on maximum opening (>2 mm) is measured with the side of a metal ruler positioned in the mid-sagittal plane. The movement of a point on the chin with regard to the side of the ruler was assessed (Figure 2b). Other assessments were not excluded. If an item scored positive, “1” was assigned, otherwise “0”, with a resulting total score of the protocol ranging from 0 to 11. We conceptualized “at least 1 positive score” as an indication for potential TMJ involvement. Composite scores were constructed for the history related items (Fh, total score ranging from 0–5) and the 6 examination related items (Fe, total score ranging from 0–6).

To test the concurrent validity of the protocol, the juvenile arthritis disease activity score (JADAS-27) [15] was used as an external reference to measure actual disease activity.

The JADAS consists of the following variables: 1) physician global rating of overall disease activity measured on a 10-cm horizontal visual analog scale (VAS) or a 21-numbered circle VAS (0: no activity; 10: maximum activity for both VAS); 2) parent/child ratings of well-being and pain, assessed on a 10-cm horizontal VAS or a 21-numbered circle VAS (0 best; 10 worst for both VAS); 3) number of active joints, assessed in 71, 27, or 10 joints (JADAS-71, JADAS-27, and JADAS-10, respectively); and 4) Westergren erythrocyte sedimentation rate (ESR), normalized to a 0–10 scale. The final result is the sum of the scores of its four components, with a global score of 0–101, 0–57, and 0–40 for the JADAS-71, JADAS-27, and JADAS-10, respectively. We used the JADAS-27, because it is more feasible than the JADAS-71, and not as limited as the JADAS-10. Moreover the TMJ’s are not included in the JADAS-27, which makes this score more appropriate for validation of the TMJ protocol than the JADAS-71. Different cut-off values have been validated for the JADAS-27. Minimal disease activity has been defined with a score below “2” for oligoarticular JIA, and below “3.8” for polyarthritis [16]. In our study, we used these two scores of disease activity to calculate cut-off points of the protocol to indicate TMJ dysfunction. The JADAS-27 was assessed during the routine clinical visit. Blood was drawn only in case of higher disease activity compared to former visits or for drug monitoring purposes. In the other cases ESR from a former visit was used (maximum three months before the examination) to calculate the JADAS-27.

### Statistical analysis

The internal consistency of the protocol was determined using Cronbach’s alpha. Inter-examiner reliability of the individual items, composite scores Fh and Fe, and the total score, as assessed by the RE and the rheumatologist, was determined using Cohen’s Kappa [17].

Correlation between the outcome variables and JADAS was tested using Pearson’s correlation. Sensitivity, specificity, positive predictive value (PPV) and negative predictive value (NPV) were established using the test result of the rheumatologist. ROC analysis was performed to calculate the cut-off point of the TMJ examination protocol. All statistical tests were 2-sided. P-values less than 0.05 were considered significant. All analyses were carried out with the statistical package SPSS version 20 for Windows (IBM corporation, USA).

## Results

A group of 76 patients, female to male ratio 2.4:1 (Table 1), was examined. Two patients were excluded after the examination because of a dental abcess and because of suspected inner ear involvement. The final group of 74 patients had a mean age of 11.9 yrs (SD 3.8) (Table 1). The rheumatologists were of opinion that the standardized TMJ evaluation could easily be included in the regular consultation time at the outpatient clinic.

The internal consistency of the protocol was 0.78. Deleting the items “asymmetry” and “mandibular retrognathism” with low agreement from the analyses, Cronbach’s alpha reached 0.85.

### Reliability of assessments: rheumatologist versus RE

Inter-observer reliability using Cohen’s Kappa of the history related items varied between 0.46 and 0.87 (Table 2). Examination related items scored between 0.25 (asymmetry, retrognathia) and 0.73 (pain on opening). The agreement between the rheumatologist and the RE regarding the total score of the TMJ protocol was .46. The mean MMO measured by the rheumatologist and the RE did not differ significantly: 46.7 mm vs. 45.8 mm (p = 0.35, Table 2). MMO measurements of the rheumatologist and the RE correlated significantly (intraclass coefficient .61, p < 0.01, Table 2), with differences less than 7 mm (smallest detectable change indicating clinical relevance) in 68 percent of the patients (Table 2). The MMO of children under 10 years old did not differ significantly from the children over 10 years old: 43.6 vs 46.3 respectively (p = 0.104, Table 1).

**Table 2:** **Reliability (Cohen’s Kappa) of the TMJ screening protocol assessments and prevalence of findings**

	**Cohen’s Kappa**	**Prevalence RE (%)**	**Prevalence Rheumatologist (%)**
*History*			
Problems in chewing	0.46	10	16
Eating slower than others	0.45	14	22
Biting hard food	0.64	14	18
Pain while eating	0.83	14	15
Limited mouth opening	0.87	11	14
*Examination*			
MMO limited*	0.42	12	15
Crepitation (audible)	0.0	5	7
Pain MMO	0.73	18	11
Deviation MMO (>2 mm)	0.31	7	15
*Inspection*			
Asymmetry	0.25	22	19
Retrognathia	0.25	16	8
TMJ involv. RE vs Rh^a^	0.46	54	51
ICC MMO _Rh vs RE_	0.61 (p < 0.01)		
Δ MMO_Rh_ - MMO_RE_ ≤7 mm [n (%)]	50 (67.6%)		
μ Δ MMO _Rh vs RE_ [mm]	0.9 (p = 0.35)		

History related items (n = 5), examination related items (n = 4), and inspection related items (n = 2) compose the screening protocol. Intraclass correlation of the rheumatologist and the reference examiner.

RE: reference examiner. ICC: intraclass coefficient.

^*^MMO limited: ≤ 35 mm (≤10 yrs); ≤ 40 mm (>10 yrs); ^a^ TMJ involvement based on the criterion ‘at least 1 positive score. μ = mean, p value Δ MMO (Students *t*-test)

The prevalence of positive clinical assessments by the RE and the rheumatologist varied between 5 and 22 percent (Table 2). In this study the maximum protocol scores were 8 and 9 assessed by the RE and the rheumatologist respectively.

The MMO measured by the rheumatologist correlated negatively with JADAS-27 (p = 0.03, Table 3). The total score of the RE and rheumatologist correlated (p < 0.01) with JADAS-27; the composite scores of the five history items and four examination items on mandibular function (asymmetry and micrognathia excluded) correlated with JADAS-27 as well (Table 3).

**Table 3:** **Pearson correlation of TMJ protocol variables and JADAS-27 for the rheumatologist and the reference examiner**

**Correlations**	**Rh r (p)**	**RE r (p)**
Pain on MMO – JADAS-27	0.21 (0.08)	0.51 (<0.01)
Pain on chewing – JADAS-27	0.47 (<0.01)	0.19 (<0.01)
Total score – JADAS-27	0.34 (0*.*003)	0.49 (<0.01)
Fh° – JADAS-27	0.36 (*<*0.01)	0.47 (<0.01)
Fe* – JADAS-27	0.26 (0.03)	0.44 (<0.01)
MMO – JADAS-27	- 0.27 (0.03)	- 0.23 (0.08)

°Fh: number of positive history scores on mandibular function; *Fe: number of positive examination scores on mandibular function. Rh: rheumatologist; RE: reference examiner; correlation r values; p: probability (Students *t*-test).

### Concurrent validity of the TMJ protocol used by the rheumatologist

The JADAS-27 was used as an external reference, available in 66 out of 74 patients. For eight patients ESR from a former visit was used. Using a cut-off point of “at least 1 positive score”, the mean JADAS-27 of the resulting patient groups was 2.0 (no TMJ involvement) vs 5.0 (TMJ involvement) (p = 0.029, Table 4). Using the cut-off point “at least 2 positive scores,” the mean JADAS in the resultant patient groups was 2.20 (no TMJ involvement) and 6.39 (TMJ involvement) (p = 0.003, Table 4). This cut-off point resulted in a potential TMJ involvement in 25 (34%) out of 74 patients (Table 4). Eleven out of these 25 patients (44%) with potential TMJ involvement had a disease activity below “3.8” (range 0.0 – 3.5). Ten of the 49 patients (20%) without potential TMJ involvement had a disease activity above “3.8” (range 3.9 – 12.4). The results of the other cut-off points are presented in Table 4.

**Table 4:** **Test characteristics of the TMJ protocol for different cut-off points; assessments by the rheumatologist**

	**no TMJ involv.**	**TMJ involv**	**Sens**	**Spec**	**PPV**	**NPV**	**p**
*Protocol score*							
*at least 1 pos score (n)*	34	40					
JADAS-27 (sd)	2.0 (2.98)	5.0 (7.13)					0.029
JADAS-27 ≥ 2			0.65	0.55	0.55	0.65	
JADAS-27 ≥ 3.8			0.65	0.51	0.38	0.77	
*Protocol score*							
*at least 2 pos scores (n)*	49	25					
JADAS-27 (sd)	2.2 (3.12)	6.39 (8.36)					0.003
JADAS-27 ≥ 2			0.47	0.78	0.64	0.63	
JADAS-27 ≥ 3.8			0.57	0.77	0.52	0.80	
*Protocol score*							
*at least 3 pos score (n)*	60	14					
JADAS-27 (sd)	2.37(3.14)	8.94 (10.2)					< 0.001
JADAS-27 ≥ 2			0.41	0.85	0.70	0.63	
JADAS-27 ≥ 3.8			0.52	0.84	0.60	0.80	
*Protocol score|*							
*at least 4 pos scores (n)*	64	10					
JADAS-27 (sd)	3.31 (5.69)	5.56 (6.26)					0.306
JADAS-27 ≥ 2			0.18	0.90	0.60	0.56	
JADAS-27 ≥ 3.8			0.22	0.90	0.50	0.72	

JADAS-27: juvenile arthritis disease activity score. Sens: sensitivity; Spec: specificity; NPV: negative predictive value; PPV: positive predictive value. p: probability (Students *t*-test).

ROC analysis indicated sensitivity and specificity of the TMJ protocol used by the rheumatologist of 0.52 and 0.80 respectively for a disease activity score of “3.8,” with the area under the curve (AUC) 0.70.

## Discussion

A screening instrument to assist rheumatologists in determining TMJ involvement was tested. The internal consistency was adequate. Cohen’s kappa values expressing reliability of the history and function related items varied between 0.42 (fair) and 0.87 (almost perfect). Concurrent validity was found to be fair to good. The rheumatologists noted that the TMJ screening could easily be included in the regular consultation time, making it applicable in standard clinical care such as follow-up.

### Reliability

The internal consistency of the protocol could be raised to 0.85 by deleting “asymmetry” and “retrognathia” with low internal correlations. However, because these variables need to be evaluated in order to assess growth disturbances and for referral to an orthodontist and/or an oro-maxillofacial surgeon, we propose that these two items should be retained in the protocol despite the lower reliability of the assessments.

A striking finding was the absence of major mandibular underdevelopment in this patient group. This finding supports a decreased future demand for surgical correction at an adult age. Using the suggested criterion of “at least 2 positive scores,” 34 percent of the patients were identified as having potential TMJ involvement (Table 4), which is close to the 42 percent detected by using contrast enhanced MRI [4].

In this study one rheumatologist examined the majority of the patients (n = 44). The other patients were examined by three other rheumatologists. Their participation is not expected to disqualify our results regarding agreement. Agreement studies indicate lower inter-examiner agreement than intra-examiner agreement. However in clinical settings the same rheumatologist will perform repeated measurements in follow-up visits and in such a scenario agreement is likely to be higher than our results.

Limitation of MMO is traditionally perceived as a key sign of TMJ involvement. Restricted mouth opening capacity is found more often in patients with JIA with radiographic signs of mandibular condyle lesions than in those without any detectable lesions [18]. In our study MMO and the JADAS-27 were negatively correlated, signifying that disease activity is associated with mandibular function (Table 3). TMJ deformity was not assessed in our study; deformity represents earlier periods of disease activity, whereas the JADAS-27 and the protocol for most of its items indicate current disease activity. Using the protocol with the criteria “at least 3 positive scores,” the mean MMO was significantly lower (6.1 mm, p = 0.023) in JIA patients with TMJ protocol scores above this cut-off value compared to JIA patients with TMJ protocol scores lower than or equal to this cut-off value, supporting construct validity. For the criterion “at least 2 positive scores,” the MMO difference trended towards significance (4.5 mm, p = 0.076). These mean differences coincide with the smallest detectable change for MMO (5–7 mm), suggesting clinical relevance [19]. Follow-up measurements of MMO in the context of the other items of the protocol allows for detecting clinical relevant changes of this parameter. The clinical relevance of discriminating restricted MMO by age can be disputed as the criterion below 10 years of age and over 10 years of age resulted in a non-significant difference of MMO (2.7 mm).

Since all patients were examined first by the rheumatologist, an order effect might have occurred. Considering the non-significant differences in MMO measurements between the rheumatologist and the RE, this effect is considered nonexistent (Table 3).

### Validity

Since the JADAS-27 is a relatively new protocol, the scores for defining minimal disease activity in JIA categories were established only recently [15,20]. A cut-off in the disease activity score of “3.8” was chosen as a good classifier for low JIA disease activity in all JIA subtypes. The lower disease activity score of “2” indicates the absence of disease activity in the oligo articular subtype alone and was considered less clinically relevant [16]. We originally conceptualized a cut-off point of “at least 1 positive score” of the TMJ protocol for TMJ involvement. The mean JADAS-27 score of the patient groups using that cut-off point was 2.0 vs 5.0 (Table 4). Because using the protocol with a cut-off point of “at least 2 positive scores” resulted in a larger contrast of the JADAS-27 (2.2 vs. 6.4), this cut-off score seems to be a better indicator to decide about TMJ involvement than ‘at least 1 positive score’. The ROC analysis supported the use of “at least 2 positive scores.” A cut-off disease activity score of “3.8” resulted in a sensitivity and specificity of 0.57 and 0.77 respectively, positive and negative predictive values being 0.52 and 0.80 respectively. The relative high percentage of oligo-articular JIA with known low level TMJ involvement in our patient group might contribute to the low sensitivity of the instrument with 3 and 4 positive scores.

We used the JADAS-27 values at the time of the examination in 66 patients. The other eight patients were in complete remission. In these eight patients a blood test wasn’t indicated on the day of examination and the JADAS-27 was calculated using ESR values originating from a period no longer than 3 months before the examination. This approach allowed for the use of the JADAS-27 in 74 patients.

### Clinical considerations

Because the screening protocol for TMJ involvement is applicable in standard clinical care it provides the rheumatologist with systematic and focused TMJ information which relates to the JIA disease activity (JADAS-27). It is a tool to detect signs and symptoms of dysfunction of the masticatory system. Diagnosing TMJ arthritis is not possible with clinical assessment only, as indeed it can exist in the absence of signs and symptoms. In order to diagnose arthritis as early as possible, we investigated the correlation between the protocol score and inflammatory activity. Ideally TMJ arthritis should be detected with 100 percent certainty and this goal is approached by MRI [21]. Measuring disease activity as leading variable for management decisions is another option, and is feasible and appropriate in the clinic practice. A positive correlation between the protocol score and the JADAS-27 offers additional support for the protocol to be used at regular intervals for identifying disease activity in the TMJ.

It is probable that MRI with contrast detects more TMJ inflammation than the screening protocol detects TMJ dysfunction. Nevertheless the clinical significance of MRI contrast enhancement is not completely known, as suggested by a recent study in which indeed TMJ MRI enhancement is compatible with a normal range of mouth opening, normal TMJ function, absence of arthritis in other joints and may be present in the context of immunosuppressive therapy [4]. The correlation of findings resulting from the protocol and MRI findings (and eventually other mandibular function variables like bite force and masticatory performance) could result in more clear indications for the use of MRI. This will be investigated in future studies. In this regard, the rheumatologic evaluation of the TMJ could start with the protocol which is associated with disease activity through the use of the JADAS-27 in a daily clinical setting and continue, if indicated, with imaging, in order to define better treatment and follow up.

Regarding disease activity, the JADAS-27 was used for correlation with the TMJ protocol, because the TMJ examination is not included in this score. If the TMJ examination would have been included in the disease activity score like in the JADAS-71, the calculated correlation with the TMJ protocol would be biased. The aim of the investigation was to test the validity of the protocol against a reference score. We chose the disease activity JADAS-27 to avoid circular reasoning. The JADAS-71 might be more suitable as disease activity score from a clinical standpoint, but methodological considerations predominated our choice for the JADAS-27.

The cut-off value for reduced MMO has been suggested throughout the literature as < 40 mm. However, there are good reasons to consider values that are age-related when examining the mandibular range of motion in children. For that reason we used two cut-off scores for children ≤ 10 years of age and > 10 years of age. Measuring MMO, like we did, indicated a wide range of MMO per age category in a population of 20709 schoolchildren [22]. The authors drew the conclusion from their study that “the use of a cut-off point for the assessment of MMO in children with potential affection of the masticatory system could not be recommended.” The authors state that even the percentiles “will not be able to solve the dilemma of interpretation of a single MMO measurement.” However they “hope that the percentile charts will become an important tool for longitudinal follow-up of children with a high risk for TMJ affections, e.g. children with JIA.” The TMJ screening protocol allows for regular measurement of mouth opening, detection of changes in the range of motion over time, as advocated by WHO for growth curves. In follow-up, change might be more informative than the mere use of a cut-off point. If a cut-off point is used, the interpretation of range of motion measurements should always be performed in the context of the patient. We did not include lateral excursions as a clinical assessment. This measure of the range of motion is more complex and thus time consuming when executed properly. Asymmetry with wide mouth opening (item number nine, Figure 2b) is highly indicative of asymmetric lateral excursions. The assessment of the vertical overbite was not included in measuring mouth opening since this measurement is time consuming as well and does not have additional clinical relevance regarding the clinical entity “mouth opening” when comparing the results of the interincisal distance between consecutive visits in the same patients. Vertical open bites between the front teeth may be indicators of condylar structural changes and as such need to be documented beyond a screening protocol.

## Conclusion

The use of the screening TMJ protocol for the rheumatologist is feasible in the everyday clinical practice. The internal consistency of the protocol was good. Several function related items regarding actual signs and symptoms of TMJ involvement in patients with JIA could be scored with moderate to almost perfect reliability. Validity using the JADAS-27 as an external reference indicates good clinical utility. Replication using the TMJ protocol in a larger patient group and the use of other external references can provide additional validity of the TMJ protocol in detecting TMJ involvement.

### Consent

Written consent was obtained from the patient and the parents for publication of Figure 2.

## Abbreviations

JIA: Juvenile idiopathic arthritis
TMJ: Temporomandibular joint
RE: Reference examiner
JADAS: Juvenile arthritis disease activity score
MMO: Maximum mouth opening
ESR: Erythrocyte sedimentation rate
